# Hormonal response after masturbation in young healthy men – a randomized controlled cross-over pilot study

**DOI:** 10.1186/s12610-021-00148-2

**Published:** 2021-12-23

**Authors:** Eduard Isenmann, Moritz Schumann, Hannah L. Notbohm, Ulrich Flenker, Philipp Zimmer

**Affiliations:** 1 Department of Molecular and Cellular Sports Medicine, Institute for Cardiovascular Research and Sports Medicine, German Sports University, Cologne, Germany; 2 Department of Fitness and Health, IST-University of Applied Sciences, Dusseldorf, Germany; 3 Department of ‘Performance and Health (Sports Medicine)’, Institute of Sport and Sport Science, Technical University Dortmund, Dortmund, Germany

**Keywords:** Sexual activity, Hormonal response, Masturbation, Testosterone, Cortisol, Free testosterone, Activité sexuelle, Réponse hormonale, Masturbation, Testostérone, Cortisol, Testostérone libre

## Abstract

**Background:**

Hormones like testosterone play a crucial role in performance enhancement and muscle growth. Therefore, various attempts to increase testosterone release and testosterone concentration have been made, especially in the context of resistance training. Among practitioners, sexual activity (coitus and masturbation) a few hours before training is often discussed to result in increases of testosterone concentration and thus promote muscle growth. However, there is no evidence to support this assumption and the kinetics of the testosterone and cortisol response after sexual activity have not been adequately investigated. Therefore, the aim of this pilot-study was to examine the kinetics of hormone concentrations of total testosterone, free testosterone and cortisol and their ratios after masturbation. In a three-arm single blinded cross-over study, the effects of masturbation with visual stimulus were compared to a visual stimulus without masturbation and the natural kinetics in healthy young men.

**Results:**

The results showed a significant between-condition difference in free testosterone concentrations. Masturbation (*p* < 0.01) and a visual stimulus (*p* < 0.05) may seem to counteract the circadian drop of free testosterone concentrations over the day. However, no statistical change was observed in the ratios between total testosterone, free testosterone and cortisol.

**Conclusions:**

It can be assumed that masturbation may have a potential effect on free testosterone concentrations but not on hormonal ratios. However, additional studies with larger sample sizes are needed to validate these findings.

**Supplementary Information:**

The online version contains supplementary material available at 10.1186/s12610-021-00148-2.

## Résumé

**Contexte:**

Les hormones comme la testostérone jouent un rôle crucial dans l’amélioration des performances et dans la croissance musculaire. Par conséquent, diverses tentatives pour augmenter la libération de testostérone et la concentration de testostérone ont été faites, en particulier dans le contexte de l’entraînement à la résistance. Chez les praticiens, l’activité sexuelle (le coït et la masturbation) quelques heures avant l’entraînement est souvent évoquée comme induisant une augmentation de la concentration de testostérone et favorisant ainsi la croissance musculaire. Il n’existe, cependant, aucune preuve à l’appui de cette hypothèse et la cinétique de la réponse de la testostérone et du cortisol après l’activité sexuelle n’a pas été étudiée de manière adéquate. Par conséquent, l’objectif de cette étude pilote était d’examiner la cinétique des concentrations hormonales de testostérone totale, de testostérone libre et de cortisol et de leurs ratios après la masturbation. Dans une étude croisée à trois bras en simple aveugle, les effets de la masturbation avec stimulus visuel ont été comparés à un stimulus visuel sans masturbation et à la cinétique naturelle chez des hommes jeunes en bonne santé.

**Résultats:**

Les résultats ont montré, pour les concentrations de testostérone libre, une différence significative entre les conditions. La masturbation (*p* < 0,01) et un stimulus visuel (*p* < 0,05) pourraient sembler contrecarrer la baisse circadienne des concentrations de testostérone libre au cours de la journée. Cependant, aucun changement statistique n’a été observé dans les ratios entre la testostérone totale, la testostérone libre et le cortisol.

**Conclusion:**

On peut supposer que la masturbation peut avoir un effet potentiel sur la testostérone libre mais pas sur les ratios hormonaux. D’autres études, avec des échantillons de plus grande taille, sont cependant nécessaires pour valider ces résultats.

## Background

Hormones such as testosterone are proposed to play a decisive role in performance development and muscle growth [1]. Testosterone is known to promote anabolic effects in skeletal muscle by stimulating protein biosynthesis and modifying fiber content [2]. In humans, 98 % of total testosterone (TT) is bound to transport proteins, such as the sex hormone-binding globulin (SHBG) and albumin [3–6], while up to 2 % of TT can be found in its biologically active form (free testosterone, FT). In contrast to TT, only FT and its metabolite dihydrotestosterone (DHT) are able to interact with the intracellular androgen receptor (AR), thereby promoting anabolic effects. In addition to testosterone, cortisol (C) also plays a decisive role in human metabolism. Cortisol is regulated by the hypothalamus-pituitary-adrenal axis [7, 8]. Studies have suggested that an increase in cortisol concentration may have a negative effect on TT concentration [9–11], however, initial studies have shown a positive correlation between FT and C levels after physical exercise [11].

Interestingly, among practitioners and on social media platforms, it has been hypothesized and discussed that sexual activity a few hours before resistance training may increase FT concentrations or the ratio of FT and C, thereby improving training adaptions, especially in terms of gaining muscle mass. However, to date there is no scientific evidence to support this assumption. When coitus and masturbation are analysed in respect to their effect on acute hormone responses, they are mainly associated with the release of endorphins, dopamine, oxytocin and prolactin [12–22]. After orgasm, various studies have shown that prolactin levels increase, whereas oxytocin and dopamine levels decrease significantly [12–19, 23]. The effect of sexual activity on the hormone concentrations of testosterone (total and free), estrogen, cortisol and luthenizing hormone (LH) has not yet been fully investigated and understood. Initial studies from the past decades have observed no changes in TT concentration during the first 60 min after masturbation or coitus [15, 24]. Furthermore, studies have focused on the effects of sexual abstinence and subsequent changes in testosterone concentration after ejaculation [25]. However, initial surveys and studies have shown that FT concentrations are influenced by both sexual activity and sexual arousal [26]. Additionally, some research has also shown that a visual stimulus acutely increases TT concentration [27–30]. To the best of our knowledge, studies on the detailed kinetics of TT, FT and C concentrations after coitus or masturbation are lacking. Thus, this pilot-study aimed to assess the influence of masturbation and/or a visual stimulus on the kinetics of TT, FT, and C concentration as well as on their ratios (TT/C; FT/TT; FT/C).

## Materials and methods

### Participants

Initially, 11 young men (age: 27.1 (3.4) years, height: 181.7 (5.2) cm, body mass: 87.7 (8.6) kg) volunteered to participate in this study, however, three had to withdraw for private reasons. All participants were healthy young men without health restrictions. They were all recruited on the campus of the German Sport University Cologne, Germany from the project coordinator within 14 days. Their health status and athletic performance were assessed by the project coordinator and medical staff. At the beginning of the examination, all participants could be declared as highly advanced strength athletes [31], without any physical, psychological or sexual dysfunction. Highly advanced strength athletes have training experience of at least three years and a performance above 150 % of body weight in back squat and 120 % of body weight in bench press [31]. Addtionally, participants trained more than four times per week. In addition, all participants were in a committed relationship and had no previous records of sexual dysfunction. Furthermore, none of the participants consumed anabolic steroids or other medications. The study design was approved by the local ethics committee and all procedures were performed in accordance with the Declaration of Helsinki. All participants were informed about the study design and gave written informed consent prior to enrolment. Participants were required to be healthy, with no use of regular medication and/or dietary supplements.

### Study design

**Fig. 1:**
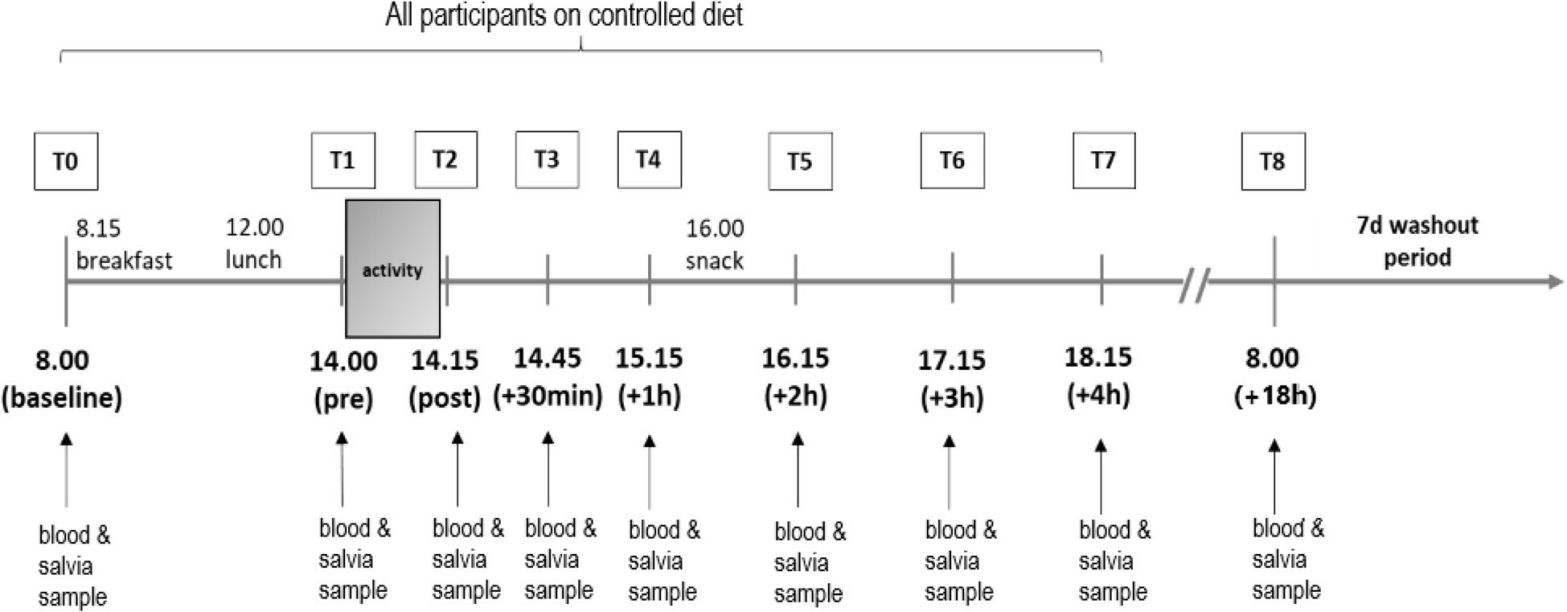
Study design. Abbreviations: d = day; h = hours; T = time point.

The study comprised of a randomized three-armed single-blind cross-over design. Changes in hormone concentrations were monitored after masturbation with visual stimulus (active group, AG), visual stimulus without masturbation (visual group, VG) and a passive setting without visual stimulus and masturbation (passive group, PG). Forty-eight hours prior to the interventions and on the examination days, all sexual and physical activity and the consumption of alcohol was prohibited. On the three intervention days, participants were also required to refrain from mental stress. All three experimental conditions were separated by a wash-out period of one week. Before each examination, participants arrived rested and fasted for the first blood and saliva sample collection (no food intake for 12 h). On the day of the intervention, food intake was standardised (supplementary material Table 1: Standardised meals with macronutrient and calorie information). In order to detect kinetic changes in hormone concentrations, 8 ml of venous blood and a saliva sample were collected at nine measurement time points (see Fig. 1).

After the second blood sample was collected (T1), participants were taken to a separate room for 15 min, where they either masturbated with the help of a visual stimulus (pornographic movie), received a visual stimulus only or received no stimulus. In the AG condition, participants were obliged to climax. In the AG and VG condition, subjects were instructed to choose a pornographic movie according to their personal needs. During the entire intervention period, the consumption of dietary supplements and medications was not allowed.

### Outcome measures

Serum testosterone concentrations were determined using the CLIA method (commercial analytical laboratory by Dr. Wisplinghoff, Cologne, Germany). Free testosterone and cortisol concentrations were determined in saliva using the salivary testosterone and cortisol ELISA kit. (Enzyme-linked Immunosorbent Assay, REF: SLV-3013 and SLV: 4635, DRG Instruments GmbH, Germany).

### Statistical analyses

All measures were transformed to their natural logarithms before data analysis [32].

The investigated measures are known to exhibit pronounced circadian rhythms. Therefore, for TT, FT, and C, trigonometric functions served as the core of a regression analysis: ln(*ρ*_*C*_ ) = *α* ⋅ cos((2 *πt*)/*τ*) + *b* ⋅ sin((2 *πt*)/*τ*), where $\rho$ represents the concentration of compound $C$ (FT, TT, C), $\tau$ is the length of the period and $t$ is the time in minutes. $\tau$ was set to 1440 min equivalent to 24 h. $a$ and $b$ are the model parameters to be estimated. These were tested for interaction with the factor activity.

For the natural logarithms of the ratios TT/C, FT/C, FT/TT trigonometric functions were less suited to depict the corresponding trends. These were modeled by 3rd order orthogonal polynomials. Again, the parameters of the polynomial terms were tested for interaction with activity.

The parameters described above, served as fixed effects in linear mixed effects models (LME). Random effects, by contrast, were consistently given by varying intercepts between individuals for each measure and by 3rd order natural splines for each level of activity within individual intercepts. This allowed for some individual variability of the trends between investigation dates.

All models initially were assumed to feature significant interactions of activity with the parameters of the trigonometric terms or with those of the polynomials, respectively. Significant interactions were set to p < 0.05. Insignificant terms were then removed stepwise. The original and the pruned models were compared by likelihood statistics. At insignificant differences the simpler model was retained [33].

R statistical language in its latest version 4.0.5 https://www.R-project.org,) was employed for statistical analysis and for creating graphics. Model fitting was performed by means of the NLME library in its latest version (version 3.1-151, https://CRAN.R-project.org/package=nlme).

## Results

**Fig. 2:**
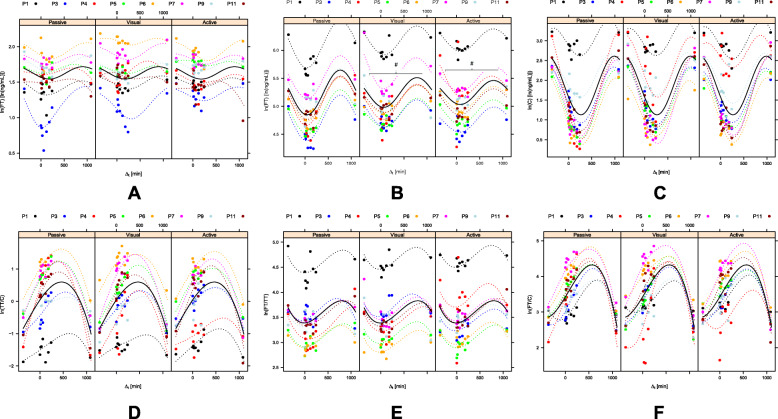
Hormone changes after masturbation with visual stimulus, only visual stimulus and passive setting. Figure 2**A-F** show the raw data (bullets), individual fits (dotted lines) and the population fits (solid lines) for each measure and grouped by activity (**A**: Total Testosterone concentration; **B**: Free Testosterone concentration; **C**: Cortisol concentration; **D**: Ratio of Testosterone/Cortisol; **E**: Ratio of Free Testosterone/Testosterone; **F**: Ratio of Free Testosterone/Cortisol). With all concentrations, both trigonometric terms are consistently required in the corresponding linear mixed effect models ($\text{p}<0.01$ respectively, $\text{F}$ statistics). The polynomial term in the models of the ratios likewise is indispensible ($\text{p}<0.001$ respectively, $\text{F}$ statistics). All significant differences were marked with #. Abbreviations: Active = masturbation with visual stimulus; C= Cortisol; FT = free testosterone; ln = logarithm; min: minutes; ng/ml = nanogram/millilitre; P = participant (P1 =participant 1 etc.); Passive: no masturbation and no visual stimulus; TT = total testosterone; Visual: visual stimulus without masturbation

With all concentrations, both trigonometric terms are consistently required in the corresponding LME ($p<0.01$ respectively, $F$ statistics). The polynomial term in the models of the ratios likewise is indispensible ($p<0.001$ respectively, $F$ statistics).

For FT the cosine term of the trigonometric function showed a significant interaction with activity $$(F=5.21,\hspace{0.17em}p<0.01)$$. $$a$$ was calculated as $$-0.392\pm 0.057$$ at no activity (estimate$$\pm$$std. error). With visual stimulus the value of $$a$$ increased by $$0.137\pm 0.061$$$$(p<0.05)$$. Following masturbation, the increase of $$a$$ was larger compared to visual stimulus, in fact by $$0.191\pm 0.061$$ as compared to inactivity (PG) ($$p<0.01$$).

In fact, the circadian drop of the concentration of FT is significantly attenuated by visual stimulation. The drop is even further attenuated by masturbation (see Fig. 2B).

None of the models of the other measures required interaction effects of the trend parameters with activity (see Fig. 2 A and 2 C-F). This applies to the concentrations as well as to all of the ratios. The modeled population curves therefore are identical for all levels of activity (see Fig. 2 A-F).

## Discussion

To the best of our knowledge, this is the first study to investigate the kinetics of masturbation on TT, FT and C concentrations and on their respective ratios.

In regards to the initial research question, it can be assumed that masturbation and visual stimulus only have an effect on FT levels. A clear difference between masturbation and the sole visual stimulus was not observed. However, there was a difference to the passive condition. This effect in FT concentration could also have a potential benefit on repeated masturbation in the course of chronic strength training. However, even the more relevant FT/C ratio was not influenced by masturbation or visual stimulation. Based on these findings, it cannot be concluded that a single sexual activity in form of masturbation prior to resistance training could potentially provoke stronger testosterone-mediated adaptions in terms of muscle growth or shift of muscle fiber content. However, it cannot be ruled out that a continuous and repeated secretion of FT in combination with strength training could potentially lead to stronger adaptations, for example in muscle growth or shift of muscle fiber content.

Apart from masturbation, the circadian rhythm of the investigated hormones and thereby the time of training may be considered as a potential modulator of muscle growth. According to our observations, resistance training should be most effective in the early evening (T7: 18.15), when the ratio of FT and C appears to be the highest (Fig. 2 F). However, several other physiological and psychological factors, such as motivation, play a crucial role in this context and should be considered when planning and conducting this kind of intervention. Of note, increased stress levels are associated with an increased cortisol release [7] and consequently negatively affect testosterone concentrations and FT/C ratio [9, 34].

This randomized cross-over pilot-trial is limited by the small sample size and the testing of FT and C concentrations in saliva, whereas TT was assessed in serum. Although strong correlations between blood and saliva levels were reported for TT, FT and C [35–37], further research should focus on one type of biomaterial with a liquid chromatography-mass spectrometry/mass spectrometry. Furthermore, a wider range of hormones should be analysed. For example, estrogen is known to interact with testosterone concentrations as well as have an effect on skeletal muscle metabolism [2, 38–42]. In addition, the inclusion of female study participants would have increased the power of this study and should be considered in future trials to determine possible differences in hormone responses after masturbation. Furthermore, no statements can be made about hormonal changes after coitus in both sexes.

## Conclusions

This pilot study for the first time demonstrates that masturbation may take effect on free testosterone levels in young males. However, relevant corresponding hormone ratios appear unchanged. Nonetheless, the phenomenon potentially may be exploited in various settings. Even though no unequivocal conclusions could be drawn concerning the effects of masturbation on all investigated endocrine parameters, the approach of systematic modulation of the endocrine system probably deserves attention. The present study may hence provide a starting point for future clinical and sports medical research. From our point of view, this will ideally cover a broader spectrum of hormones and the investigation of corresponding interactions. Moreover, the phenomenon should be investigated in female subjects as well.

## Supplementary information

**Additional file 1**

## Data Availability

The raw data can be viewed upon reasonable request.

## Abbreviations

AG: active group
AR: androgen receptor
C: cortisol
DHT: dihydrotestosterone
FT: free testosterone
LH: luthenizing hormone
LME: linear mixed effects models
PG: passive group
SHBG: sex hormone-binding globulin
TT: total testosterone
VG: visual group
