# Supplementary material for: Hormonal response after masturbation in young healthy men – a randomized controlled cross-over pilot study
Source: Basic Clin Androl. 2021 Dec 23;31:32. doi: 10.1186/s12610-021-00148-2 (PMC8697462; doi:10.1186/s12610-021-00148-2)
Supplement: Supplementary file 1 — Additional file 1 [file 12610_2021_148_MOESM1_ESM.docx]

Table 1: Standardised meals with macronutrient and calorie information

|  | **Time point** | | | | | **Food** | **Quantity** | **Proteins** | **Carbo-**  **hydrates** | **Fat** | **Calories** |
| --- | --- | --- | --- | --- | --- | --- | --- | --- | --- | --- | --- |
|  | **8.00 Breakfast** | | | | | oat flakes (JA oat flakes) | 60g | 9.1g | 54.3g | 4.4g | 349.8 kcal |
|  |  |  |  |  |  | banana (Chiquita) | 115g  (one piece) |  |  |  |  |
|  |  |  |  |  |  | Honey (Langnese) | 5g |  |  |  |  |
|  |  |  |  |  |  | water | individual |  |  |  |  |
|  |  |  |  |  |  |  |  |  |  |  |  |
|  | **12.00 Lunch**  (prepmymeal) | | | | | Red lentils  Rice  Green beans  Currysauce |  | 30.0g | 120.0g | 2.0g | 650 kcal |
|  | **16.00 Snack** | | | | | banana (Chiquita) | 115g  (one piece) | 1.2g | 25.3g | 0.2g | 110.4 kcal |
|  |  | | | | | Flapjack (Hafervoll) | 65g  (one bar) | 4.8g | 31g | 19g | 324 kcal |
|  | **Total** | | | | |  |  | **45.1** | **230.6** | **25.6** | **1434.2 kcal** |
